# The Study of the Intercellular Trafficking of the Fusion Proteins of Herpes Simplex Virus Protein VP22

**DOI:** 10.1371/journal.pone.0100840

**Published:** 2014-06-23

**Authors:** Xiaodong Xue, Jianhua Huang, Huishan Wang

**Affiliations:** 1 Department of Cardiovascular Surgery, General Hospital of Shenyang Military Area Command, Shenyang, Liaoning, China; 2 Department of Cardiothoracic Surgery, Ningxia People’s Hospital, Yinchuan, Ningxia, China; University-Hospital of Parma, Italy

## Abstract

**Background:**

Genetic modifications can improve the therapeutic efficacy of mesenchymal stem cell (MSC) transplantation in myocardial infarction. However, so far, the efficiency of MSC modification is very low. Seeking for a more efficient way of MSC modification, we investigated the possibility of employing the intercellular trafficking capacity of the herpes simplex virus type-1 tegument protein VP22 on the enhancement of MSC modification.

**Methods:**

Plasmids pVP22-myc, pVP22-EGFP, pEGFP-VP22, pVP22-hBcl-xL and phBcl-xL-VP22 were constructed for the expressions of the myc-tagged VP22 and the fusion proteins VP22-EGFP, EGFP-VP22, VP22-hBcl-xL and hBcl-xL-VP22. MSCs were isolated from rat bone marrow and the surface markers were identified by Flowcytometry. COS-1 cells were transfected with the above plasmids and co-cultured with untransfected MSCs, the intercellular transportations of the constructed proteins were studied by immunofluorescence. The solubility of VP22-hBcl-xL and hBcl-xL-VP22 was analyzed by Western blot.

**Results:**

VP22-myc could be expressed in and spread between COS-1 cells, which indicates the validity of our VP22 expression construct. Flowcytometry analysis revealed that the isolated MSCs were CD29, CD44, and CD90 positive and were negative for the hematopoietic markers, CD34 and CD45. The co-culturing and immunofluorescence assay showed that VP22-myc, VP22-EGFP and EGFP-VP22 could traffic between COS-1 cells and MSCs, while the evidence of intercellular transportation of VP22-hBcl-xL and hBcl-xL-VP22 was not detected. Western blot analysis showed that VP22-hBcl-xL and hBcl-xL-VP22 were both insoluble in the cell lysate suggesting interactions of the fusion proteins with other cellular components.

**Conclusions:**

The intercellular trafficking of VP22-myc, VP22-EGFP and EGFP-VP22 between COS-1 cells and MSCs presents an intriguing prospect in the therapeutic application of VP22 as a delivery vehicle which enhances genetic modifications of MSCs. However, VP22-hBcl-xL and hBcl-xL-VP22 failed to spread between cells, which are due to the insolubility of the fusion protein incurred by interactions with other cellular components.

## Introduction

Cell transplantation has emerged as a promising therapeutic approach for the restoration of heart function after myocardial infarction. Various cell types, including embryonic stem cells [1], cardiac stem cells [2], skeletal myoblasts [3]–[5], smooth muscle cells [6], [7], bone marrow cells [8] and hematopoietic stem cells [9] etc. have been studied in cell transplantation and were demonstrated to be useful in the replacement of the injured myocardium and in the improvement of angiogenesis and heart functions.

Bone marrow mesenchymal stem cells (MSCs) are self-renewing, multipotent precursors of non-hematopoietic stromal tissues [10]–[12]. Under appropriate conditions, MSCs can be induced to differentiate into multiple cell lines, which includes osteoblasts, chondrocytes, adipocytes [10], [13], skeletal muscle cells [14], cardiomyocytes [15], [16] and neural cells [17]. MSCs were demonstrated to be able to promote angiogenesis and the survival of ischemic cardiomyocytes through the paracrine production of various angiogenic and growth factors [18], [19]. Furthermore, it was shown that MSCs are immunosuppressive favoring the inhibition of inflammatory responses and the future fibrosis of the injured heart tissue [20], [21]. MSCs can be easily isolated from the bone marrow or adipose tissue and expanded in vitro based on their ability to adhere to culture dishes. Therefore, MSCs appear to be an appealing cell source for transplantation therapy in myocardial infarction [22], [23].

However, within the first few days after transplantation, the low survival rate of MSCs incurred from the deleterious microenvironment of ischemia, inflammatory response and proapoptotic factors severely holds back the therapeutic effects on the cardiomyocytes restoration [16], [24]. Thus, it is necessary to reinforce MSCs to improve the efficacy of cell therapy. Evidences have demonstrated that genetic modification of MSCs with survival [25]–[27] or anti-apoptotic [28] genes can improve the viability of the transplanted MSCs and results in a better homing of MSCs into the ischemic microenvironment thus enhancing the cardiac functional recovery after acute myocardial infarction.

So far, viral and non-viral methods were both used for MSC genetic modification. Viral vectors had been proved to be efficient in MSC genetic manipulation, however, the complicated production procedure, the risks of host immunogenicity and tumorigenicity impedes the wide use of viral vectors [29]–[33]. Non-viral vectors, such as plasmids, present certain advantages over viral vectors with simpler large scale production, less likely to induce immune responses and less toxic [33]. However, the gene transfer efficiencies of plasmids are usually too low to meet the therapeutic purposes. Efforts had been made to increase the efficiency of non-viral gene delivery [34]–[37]. In this study, we focused on another way to enhance the plasmid-mediated MSC modification. In short, if the gene product of MSC modification could traffic between cells on its own, then the bio-effects of the modified genes could be spread among MSCs and/or among MSCs and cardiomyocytes after transplantation, which could substantially enhance the efficiency of MSC modification. And obviously, it would be no necessary to obtain a high efficiency for plasmid transfection. Therefore, it is intriguing to promote the extracellular propagation of modified gene products in MSCs after transplantation for a better therapeutic outcome.

The UL49 gene of herpes simplex virus type-1 (HSV-1) encodes a 301 amino acids tegument protein VP22. It was demonstrated that VP22 has a remarkable ability of intercellular trafficking via a Golgi independent pathway [38]. The typical spread pattern of VP22 is from the cytoplasm of an expressing cell into the neighboring cells and accumulation in the nucleus. This significant discovery has inspired a series of efforts to apply VP22 as a therapeutic intercellular delivery vehicle by fusing different exogenous genes to it. So far, VP22 has been successfully expressed and trafficked in more than 10 cell lines and several VP22 fusion proteins have been successfully delivered between cells without disturbing the functions of the fused genes [39]–[43]. The VP22 mediated gene delivery precludes the need of using viral vectors for a high modification efficacy. Therefore, VP22 could be a powerful tool for the enhancement of MSCs modification.

Bcl-xL and Bcl-2 are both members of the Bcl-2 protein family. They are important regulators of cell apoptotic pathway [44]. It was demonstrated that the adenoviral mediated expression of human *Bcl-xL* (*hBcl-xL*) gene in rat heart can inhibit the apoptosis of ischemic cardiomyocytes after myocardial infarction and can prolong the cold preservation time period for cardiac transplants [45], [46]. And also, It was reported that the over expressed *Bcl-2* gene can protect MSCs against apoptosis under hypoxic conditions both in vitro and in vivo and the transplantation of the *Bcl-2* gene engineered MSCs may improve heart functional recovery after acute myocardial infarction [28]. Therefore, it is plausible to speculate that the over expression of *Bcl-xL* gene in MSCs might also improve the viability of the transplanted MSCs and help to restore heart function after myocardial infarction. Furthermore, it is not known whether the fusion protein of VP22 and hBcl-xL could traffic among MSCs or between MSCs and cardiomyocytes. If this is the case, the expression of the fusion protein of VP22 and hBcl-xL in MSCs might protect both the transplanted MSCs and the ischemic cardiomyocytes against apoptosis during the early stage of the post-transplant period and hence obtain a better curative effect on myocardial infarction.

In this study, we constructed plasmids for the expressions of the VP22-myc and the fusion proteins VP22-EGFP, EGFP-VP22, VP22-hBcl-xL and hBcl-xL-VP22. To investigate the intercellular propagation of VP22 and the fusion proteins, COS-1 cells were transfected with the above plasmids and co-cultured with rat bone marrow MSCs. Twenty four hours after co-culturing, we found that VP22-myc, VP22-EGFP and EGFP-VP22 could spread between COS-1 cells and MSCs. However, we did not observe any intercellular trafficking of VP22-Bcl-xL and hBcl-xL-VP22. We further checked the solubility of VP22-Bcl-xL and hBcl-xL-VP22 expressed by COS-1 cells and found that the fusion proteins were in the insoluble part of the whole cell lysate. This insoluble character could explain the lack of intercellular delivery of VP22-Bcl-xL and hBcl-xL-VP22. Nonetheless, the observed intercellular trafficking of VP22-myc, VP22-EGFP and EGFP-VP22 between COS-1 cells and MSCs could lead to an intriguing therapeutic application of VP22 as an intercellular delivery vehicle for the enhancement of MSC modification and thereby to obtain a better therapeutic effect on myocardial infarction.

## Materials and Methods

All animals were treated according to the Guide for the Care and Use of Laboratory Animals published by the US National Institutes of Health (NIH Publication No. 85-23, revised 1996) and all animal protocols were approved by the Animal Care and Use Committee of the Shenyang Northern Hospital, Shenyang City, China.

### Cells

COS-1 and HeLa cells were all purchased from American Type Culture Collection (ATCC, Rockefeller, MD, USA) and maintained in Dulbecco’s modified Eagle’s medium (DMEM; Gibco, Grand Isle, NY, USA) containing 10% fetal bovine serum (Gibco, Mulgrave, Victoria, Australia). MSCs were isolated and cultured from bone marrow of Sprague-Dawley rats. Briefly, Sprague-Dawley rats of 3 month old (Beijing Experimental Animal Center, Beijing, China) were euthanized by overdose of choral hydrate. Femurs and tibias were dissected and flushed with 30 ml phosphate buffered saline (PBS) containing 100 U/ml heparin using a 21-gauge needle. The isolate was centrifuged at 200×g for 5 minutes, washed and resuspended into 10 ml Low-glucose Dulbecco’s modified Eagle’s medium (DMEM-LG; Gibco, Grand Isle, NY, USA) containing 20% fetal bovine serum (Gibco, Mulgrave, Victoria, Australia), 2 mM L-glutamine, 100 U/ml penicillin and 100 µg/ml streptomycin. The Cells were plated into 100 mm plastic culture dishes (Beckton Dickinson, San Jose, CA, USA) and incubated at 37°C in 5% CO2 and 95% humidity for 72 hours without checking and then the cultures were washed twice consecutively with PBS and cultured in complete medium. The medium was changed three times per week and the nonadherent hematopoietic cells were completely washed out after 4 changes of medium. At 80% confluence, cells were treated with 0.25% trypsin and passaged at a ratio of 1∶3. The third passage (P3) of MSCs was used in the study.

### Flowcytometry

Cells were rinsed twice with PBS, trypsinized and centrifugation at 200×g for 5 min, and then resuspended in 500 µL PBS. Approximately 5×10^5^ cells per 100 µL were labeled with primary mouse antibodies against rat CD29, CD34, CD44, CD45 and CD90 at 4°C for 30 min and washed. The labeled cells were analyzed using a flow cytometer (Beckton Dickinson, San Jose, CA, USA). The antibodies used in this experiment were: CD29- FITC, CD34-FITC, CD44- FITC, CD45- FITC and CD90- FITC (Beckton Dickinson, San Jose, CA, USA). Mouse IgG1-FITC (Beckton Dickinson, San Jose, CA, USA) was used as an isotype control.

### Plasmids

The artificially synthesized coding sequence of *VP22* (Gene ID: 2703417) (Takara Biotechnology, Dalian, Liaoning, China) was maintained in plasmid pBackZero-T (Takara Biotechnology, Dalian, Liaoning, China). To generate the eukaryotic expression plasmid pVP22-myc, the 902 bp PCR product of the *VP22* coding sequence from plasmid pBackZero-T was flanked with a HindIII and a *Eco*RI restriction site at the 5′ and 3′ end respectively and was inserted in frame into the *Hin*dIII/*Eco*RI cut plasmid pcDNA3.1/*myc*-His B (Invitrogen, Carlsbad, CA, USA) under the control of the human cytomegalovirus (CMV) promoter, a Kozak consensus translation initiation site was added immediately before the start codon of *VP22* coding sequence to further increase the translation efficiency in eukaryotic cells. For the expressions of fusion protein VP22-EGFP and EGFP-VP22, plasmids pVP22-EGFP and pEGFP-VP22 were constructed by subcloning the *BgL*II/*Bam*HI flanked fragment of the *VP22* coding sequence in frame into the equivalently digested plasmids pEGFP-N1 and pEGFP-C1 (Clontech, Heidelberg, Germany), both plasmids contain a red-shifted variant of wild-type *GFP* (*EGFP*) and a Kozak consensus site, an additional Kozak site was added before the start codon of *VP22* coding sequence as mentioned above. To generate plasmid phBcl-xL, the cDNA of *hBcl-xL* was subcloned in frame into plasmid pcDNA3.1/*myc*-His B (Invitrogen, Carlsbad, CA, USA). To produce the fusion protein of VP22-hBcl-xL, plasmid pVP22-hBcl-xL was generated by subcloning the *Eco*RI flanked PCR product of *hBcl-xL* cDNA into the equivalently cut plasmid pVP22-myc in frame. To generate the fusion protein of hBcl-xL-VP22, plasmid phBcl-xL-VP22 was constructed by subcloning the *Hind*III flanked PCR product of *hBcl-xL* cDNA into the equivalently cut plasmid pVP22-myc in frame.

### Transfection

Cells were seeded at 5×10^4^ cells/well into 24-well plates 24 hours before transfection. The transfection was performed using the lipid-based transfection reagent POLOdeliverer 3000 (R&S, Shanghai, China) and the transfection conditions were optimized according to the manufacture’s protocol. In brief, for each well, 0.5 µg of plasmid DNA and 1 µl of POLOdeliverer 3000 were diluted separately in 25 µl Opti-MEMI Reduced Serum Medium (Invitrogen, Carlsbad, CA, USA) without serum. The dilutions were combined and incubated for 20 minutes, then added into each well containing 0.5 ml culture medium without antibiotics. Eight hours after transfection, medium was changed. To transfect cells in 6-well plate, the amounts of cells, plasmid DNA, transfection reagent and medium were scaled up in proportion to the relative surface area.

### Co-culturing and Immunofluorescence Assay

COS-1 cells were transfected with pVP22-myc, pVP22-EGFP, pEGFP-VP22, pVP22-hBcl-xL and phBcl-xL-VP22 separately. Twenty-four hours after transfection, COS-1 cells were trypsinized, counted and resuspended with HeLa cells or MSCs in ratio of 1∶20. The mixed cells were seeded on cover slips and cultured for 24 hours. Cells were rinsed with PBS and fixed in 4% Paraformaldehyde for 15 minutes, then washed 2 times in PBS and treated in 0.5% TritonX-100 for 8 minutes for membrane permeabilization. The permeablized cells were blocked with 4% BSA in PBST for 1 hour at room temperature and incubated with the first antibody at 4°C overnight, then washed and incubated with the second antibody for 1 hour at room temperature. The stained cells were rinsed 5 times with PBS and mounted with ProLong Gold antifade reagent with DAPI (Invitrogen, Carlsbad, CA, USA).

To make a better distinction between COS-1 cells and MSCs, the transfected COS-1 cells were also co-cultured with labeled MSCs. For MSCs labeling, when the cells reached 50% confluence, 5-bromo-2-deoxyuridine (BrdU) (Sigma, Saint Louis, MO, USA) was added into the culture medium at a final concentration of 40 µM and cultured at 37°C overnight. The BrdU labeled MSCs were washed in PBS for 3 times and co-cultured with the transfected COS-1 cells as mentioned above. After staining, the cells were mounted with ProLong Gold antifade reagent (Invitrogen, Carlsbad, CA, USA).

The first antibodies used were: mouse monoclonal anti-myc antibody (Invitrogen, Carlsbad, CA, USA), rabbit polyclonal anti-SV40 T Ag antibody (Santa Cruz, Dallas, TX, USA), rabbit polyclonal anti-Bcl-xL antibody (Cell signaling Tech, Boston, MA, USA) and rat monoclonal anti-BrdU antibody (Abcam, Cambridge, Cambs, UK); the second antibodies used were: cy3 conjugated goat-anti-mouse lgG (Proteintech, Chicago, IL, USA), FITC conjugated goat-anti-rabbit IgG (Proteintech, Chicago, IL, USA) and DyLight 405 conjugated goat-anti-rat IgG (Abbkine, Redlands, CA, USA).

### Imaging

Microscopic pictures were captured under a Zeiss LSM 710 inverted confocal microscope (Zeiss, Oberkochen German).

### Western Blot Analysis

Cos-1 cells were harvested 48 h after transfection and lysed either in RIPA lysis buffer [50 mM Tris-HCl (pH 7.4), 150 mM NaCl, 1% Triton X-100, 1% sodium deoxycholate, 0.1% sodium dodecyl sulfate, 2 mM sodium pyrophosphate, 25 mM β-glycerophosphate, 1 mM EDTA, 1 mM Na3VO4, 0.5 ug/ml leupeptin] or in NP40 lysis buffer [50 mM Tris (pH 7.4), 150 mM NaCl, 1% NP-40, 1 mM henylmethylsulfonyl fluoride, 2 mM sodium pyrophosphate, 25 mM β-glycerophosphate, 1 mM EDTA, 1 mM Na3VO4, 0.5 ug/ml leupeptin]. The cell lysates in NP-40 lysis buffer were further sonicated and centrifuged to obtain fractions containing soluble supernatants and insoluble pellets. The protein concentration of the samples was determined using Pierce BCA Protein Assay Kit (Thermo Scientific, Rockford, IL, USA). Proteins were resolved by 10% sodium dodecyl sulfate polyacrylamide gel electrophoresis (SDS-PAGE) and transferred to 0.22 µm PVDF membrane (EMD Millipore, Billerica, MA, USA) and probed with the first antibodies at 4°C overnight, then washed and incubated with horseradish peroxidase conjugated secondary antibodies for 1 hour at room temperature. Reactive bands were developed and enhanced by SuperSignal West Pico chemiluminescent detection reagents according to the instructions of the manufacturer (Thermo Scientific, Rockford, IL, USA). The first antibodies used were: mouse monoclonal anti-myc antibody (Invitrogen, Carlsbad, CA, USA), rabbit polyclonal anti-Bcl-xL antibody (Cell signaling Tech, Boston, MA, USA), mouse monoclonal anti-β-Tubulin antibody (Sungene Biotechnology, Tianjin, China) (loading control).

## Results and Discussion

### The Expression of VP22 in COS-1 Cells

**Figure 1:**
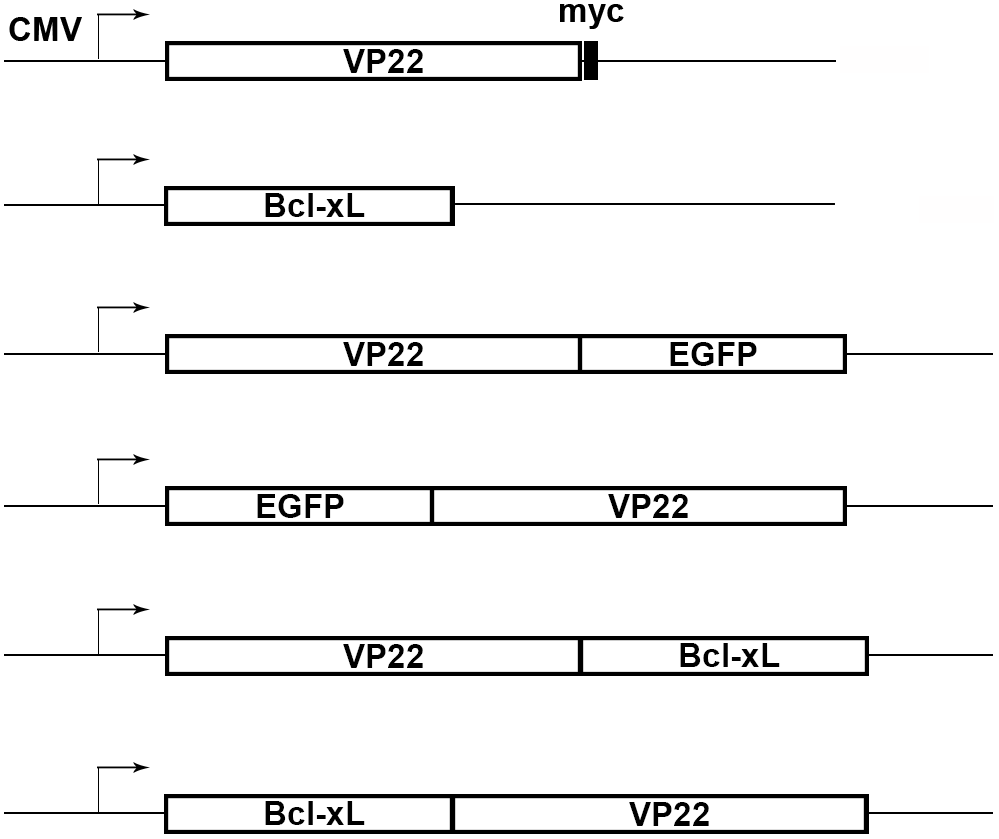
Schematic representation of the expression constructs. Schematic representation of the regions of the expression plasmids encoding myc-tagged VP22, wild type Bcl-xL and fusion proteins VP22-EGFP, EGFP-VP22, VP22-hBcl-xL and hBcl-xL-VP22. All expressions were driven by CMV promoter.

**Figure 2:**
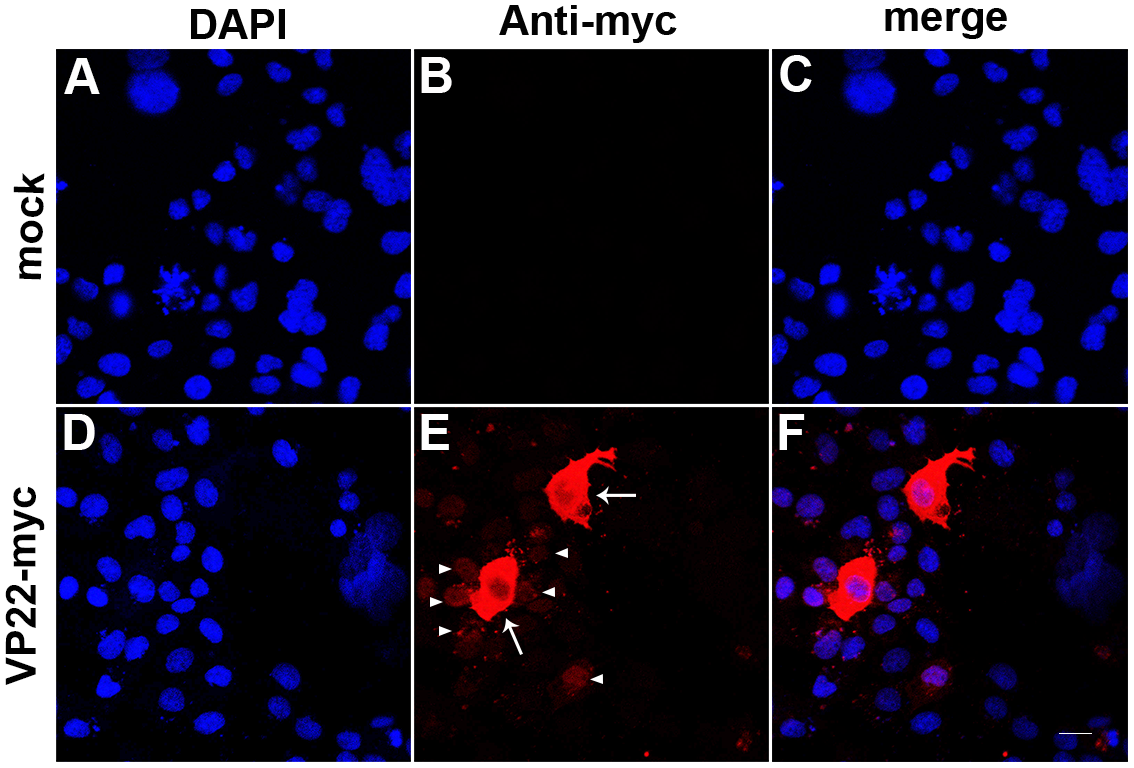
The expression of VP22 in COS-1 cells. COS-1 cells were transfected with 0.5 µg pEGFP-N1 as mock or pVP22-myc and immunostained with anti-myc antibody 48 h after transfection. The anti-myc antibody showed no cross-reaction with mock transfected cells (A–C). The myc-tagged VP22 could be expressed in COS-1 cells (D–F). An intercellular transportation of VP22 protein could be inferred from the specific VP22 protein intercellular distribution: higher cytoplasmic expression in the center cells (arrows) and lower nucleic expression in the surrounding cells (arrow heads) (E). Scale bar represents 20 µm.

To determine a more efficient way to genetically modify MSC using plasmid vectors, we employed the intercellular trafficking capacity of the HSV-1 tegument protein VP22 to enhance the intercellular transportation of the modified gene products. Firstly, we constructed plasmid pVP22-myc for wild type VP22 expression driven by the CMV promoter region (Figure 1). In this plasmid, the C-terminal myc tag works for the detection of full length expression of VP22 protein. Since wild type VP22 have been reported to be successfully expressed and delivered between COS-1 cells [38], [47], we first checked the validity of our VP22 expression construct on the expression of VP22 protein in COS-1 cells. COS-1 cells seeded on cover slips were transfected with 0.5 µg pEGFP-N1 as mock or with 0.5 µg pVP22-myc and the expression of VP22-myc was detected by immunofluorescent staining using anti-myc antibody 48 h after transfection. In control experiments the anti-myc antibody showed no cross reaction with mock transfected cells (Figure 2, A–C). The results showed that the myc-tagged VP22 could be expressed in COS-1 cells (Figure 2 D–F). And, an intercellular trafficking of the myc-tagged VP22 protein could also be inferred from the specific VP22 protein intercellular distribution: higher cytoplasmic expression in the center cells (Figure 2 E, arrows) and lower nucleic expression in the surrounding cells (Figure 2 E, arrow heads), which was concordant with the previously published data [38].

### Isolation and Characterization of Rat Bone Marrow MSCs

**Figure 3:**
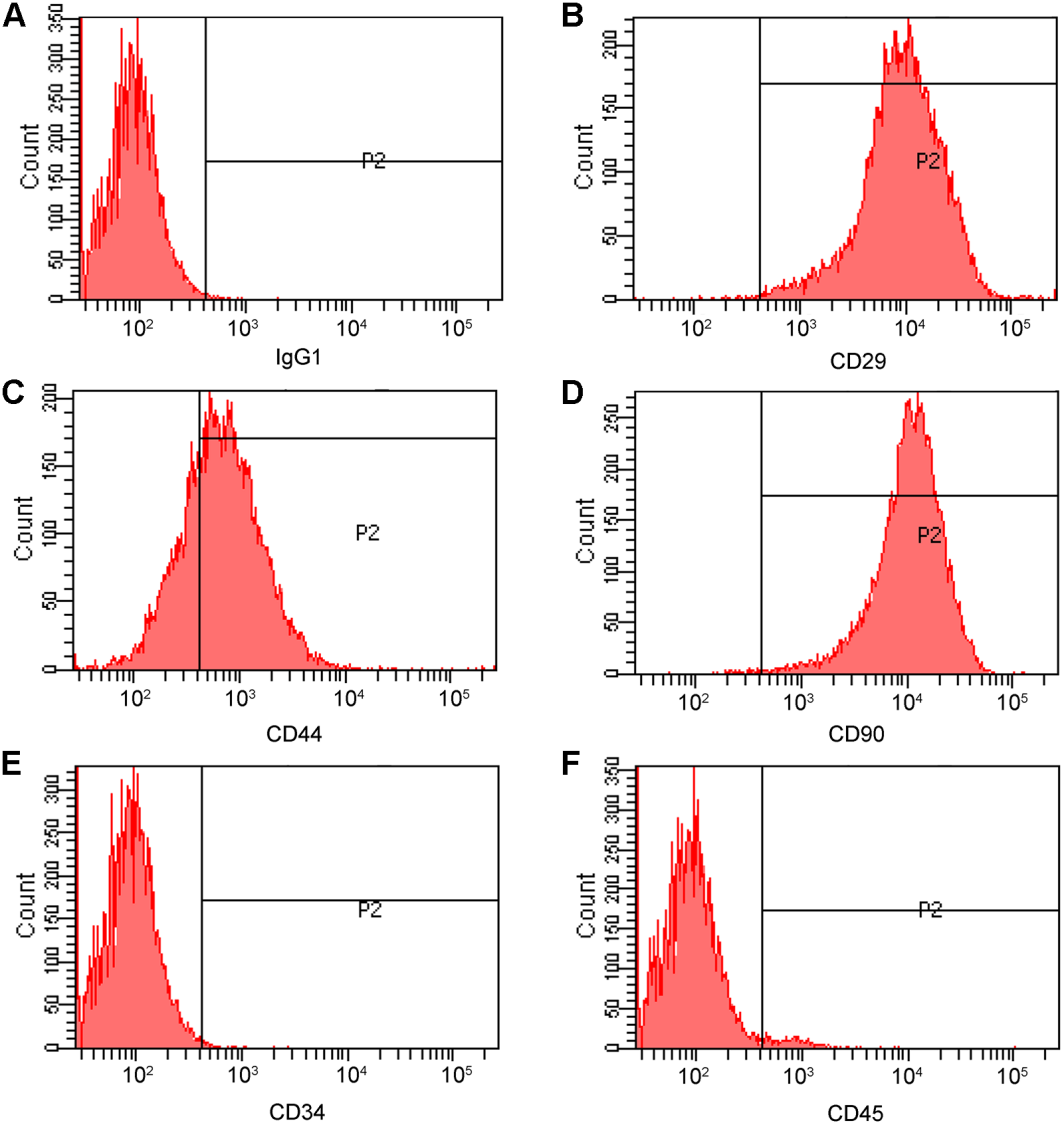
The surface marker expression of rat bone marrow MSCs. The expressions of selected surface markers of P3 MSCs were analyzed by Flowcytometry. Mouse IgG1 was used as an isotype control (A); in bone marrow MSCs, CD29, CD44, and CD90 were highly expressed (B–D) while the markers for hematopoietic stem cells, CD34 and CD45, were not expressed (E and F).

VP22 have been demonstrated to be able to travel between different cell lines, for example, between COS-1 cells and HeLa cells [38]. However, it is not known whether VP22 could traffic between COS-1 cells and MSCs. So we checked the ability of our expressed VP22-myc protein to traffic between COS-1 cells and MSCs. Firstly, we isolated MSCs from rat bone marrow according to the previously reported method [48]. Briefly, whole bone marrow was flushed off the long bones and directly plated on culture dishes. Since the dish adhering manner of MSCs was different from those of other bone marrow cell lines (hematopoietic stem cells, for example), MSC population could be purified simply by repeated medium changing when the other cell species be washed away. Monolayer rat bone marrow MSCs displayed a homogenous spindle-shaped morphology lining up in a vortex-like pattern and this morphology was maintained during the subsequent passages. The surface marker expressions of the P3 bone marrow MSCs were identified by FCM analysis. It was shown that CD29, CD44, and CD90 were highly expressed in MSCs (Figure 3, B–D) while the markers for hematopoietic stem cells, CD34 (Figure 3, E) and CD45 (Figure 3, F), were not expressed. The results from FCM analysis were confirmed by immunocytochemistry (data not shown).

### Intercellular Trafficking of VP22 between COS-1 Cells and MSCs

**Figure 4:**
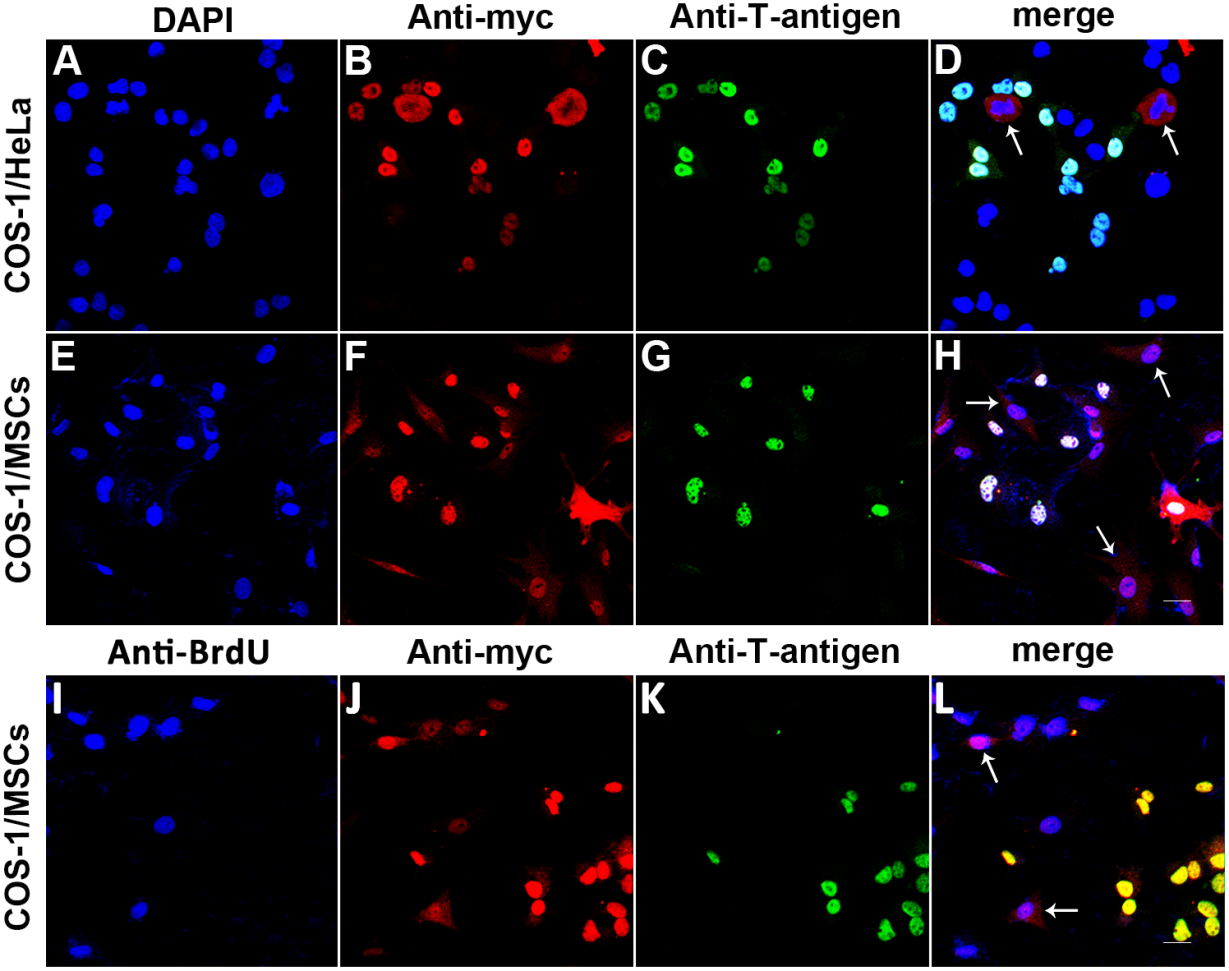
Intercellular trafficking of VP22-myc between COS-1 cells and MSCs. COS-1 cells transfected with 0.5 µg pVP22-myc DNA were trypsinized 24 hours after transfection and were co-plated with untransfected HeLa cells (A–D), MSCs (E–H) or BrdU labeled MSCs (I–L) at a ratio of 1∶20 respectively. The co-cultured cell populations were incubated for another 24 hours and were stained with anti-myc (B, F and J), anti-T-antigen (C, G and K) and anti-BrdU (I) antibodies. VP22-myc was detected not only in T-antigen positive COS-1 cells but also in the T-antigen negative HeLa cells (arrows in D) and in the T-antigen negative and/or BrdU positive MSCs (arrows in H and L), demonstrating the spread of VP22 protein. Scale bar represents 20 µm.

COS-1 cells transfected with pVP22-myc expression plasmid were trypsinized 24 hours after transfection and were co-plated with the untransfected P3 rat bone marrow MSCs at a ratio of 1∶20 (Figure 4 E–H). The transfected COS-1/HeLa cell co-culturing (Figure 4 A–D) served as a positive control. The co-cultured cell populations were incubated for another 24 hours and were double stained with anti-myc and anti-T-antigen antibodies. Since COS-1 cells express T-antigen uniformly, the VP22 primary expressing COS-1 cells would be labeled with both antibodies and be recognized easily, while the recipient MSCs would be marked with a single colored anti-myc signal. In agreement with previously reported data [38], VP22-myc was detected not only in T-antigen positive COS-1 cells but also in the T-antigen negative HeLa cells (Figure 4 D, arrows) and MSCs (Figure 4 H, arrows).

**Figure 5:**
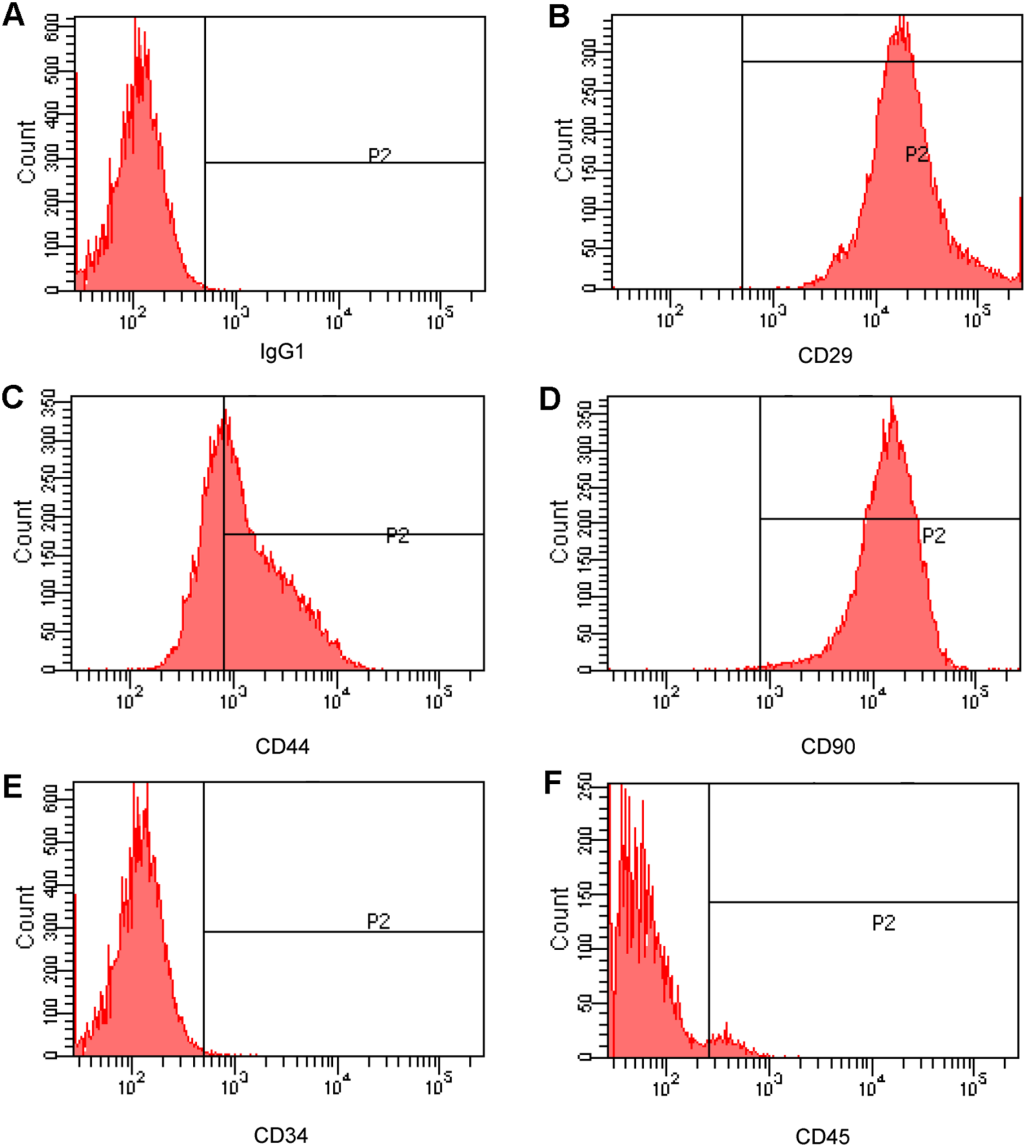
The surface marker expression of BrdU labeled bone marrow MSCs. The expressions of selected surface markers of BrdU labeled MSCs were analyzed by flowcytometry. Mouse IgG1 was used as an isotype control (A); in bone marrow MSCs, CD29, CD44, and CD90 were highly expressed (B–D) while the markers for hematopoietic stem cells, CD34 and CD45, were not expressed (E and F).

In order to make a better distinction between the VP22 fusion protein producer cells and the recipient cells in the co-culture experiment, we labeled MSCs with 5-bromo-2-deoxyuridine (BrdU). And, immunophenotypic characterization of the BrdU labeled MSCs using flow cytometry analysis showed that the expression patterns of the surface markers were similar to those in the primary cultured MSCs (Figure 5). The labeled MSCs were co-plated with the transfected COS-1 cells (Figure 4 I–L) as mentioned above and incubated for 24 hours. The co-cultured cell populations were triple stained with anti-myc, anti-T-antigen and anti-BrdU antibodies. Similarly, VP22-myc was detected not only in T-antigen positive COS-1 cells but also in the T-antigen negative/BrdU positive MSCs (Figure 4 L, arrows).

So, the results demonstrated that the myc-tagged VP22 could travel between COS-1 cells and MSCs.

### Intercellular Trafficking of Fusion Proteins VP22-EGFP and EGFP-VP22 between COS-1 Cells and MSCs

**Figure 6:**
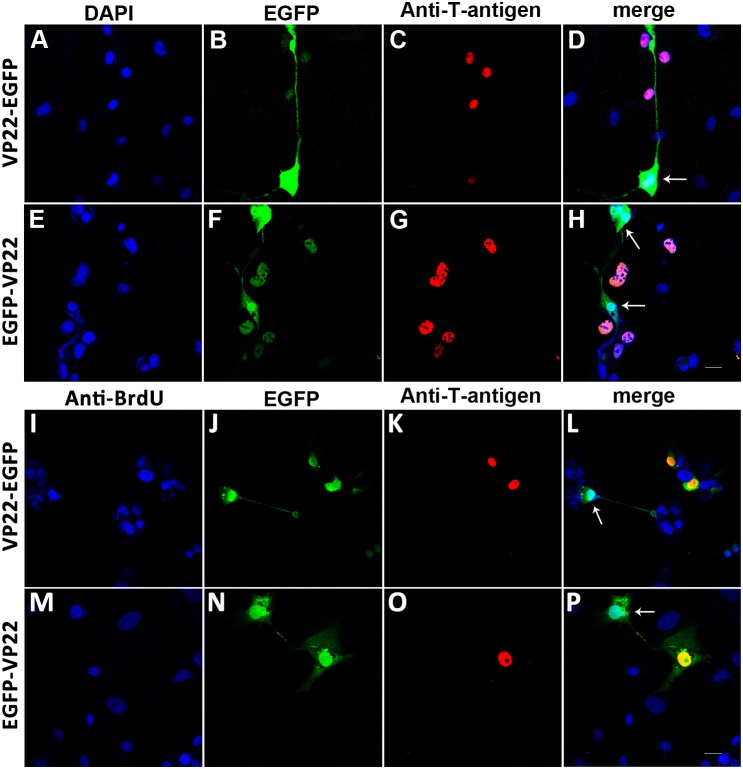
Intercellular trafficking of fusion proteins VP22-EGFP and EGFP-VP22 between COS-1 cells and MSCs. COS-1 cells transfected with 0.5 µg pVP22-EGFP (A–D and I–L) and pEGFP-VP22 (E–H and M–P) DNA were trypsinized 24 hours after transfection and were co-plated with MSCs (A–H) or BrdU labeled MSCs (I–P) at a ratio of 1∶20. The co-cultured cell population were incubated for another 24 hours and were stained with anti-T-antigen (C, G, K and O) and anti-BrdU (I and M) antibodies. EGFP signals were detected not only in T-antigen positive COS-1 cells but also in the T-antigen negative and/or BrdU positive MSCs (arrows in D, H, L and P), demonstrating the spread of VP22-EGFP and EGFP-VP22 fusion proteins. Scale bar represents 20 µm.

The spread of VP22 between COS-1 cells and MSCs indicates the possibility of using VP22 as a vehicle for the enhancement of the intercellular delivery of gene products for MSCs modification. To this vehicle, further investigations are necessary to confirm whether the intercellular trafficking capacity of VP22 between COS-1 cells and MSCs could be retained when it is fused with other genes. As we know, several VP22 fusion proteins, including VP22-EGFP and EGFP-VP22, have been successfully expressed and delivered between different cell lines [38]. Thus, we expressed fusion proteins VP22-EGFP (Figure 6 A–D) and EGFP-VP22 (Fig. 6 E–H) in COS-1 cells by transfecting the cells with 0.5 µg pVP22-EGFP or pEGFP-VP22 (Figure 1) respectively, then the transfected COS-1 cells were trypsinized 24 hours after transfection and were co-plated with untransfected MSCs at a ratio of 1∶20. The co-cultured cell populations were incubated for another 24 hours and were immunostained with anti-T-antigen antibody. The green colored signals of EGFP were detected not only in T-antigen positive COS-1 cells but also in the T-antigen negative MSCs (Figure 6 D and H, arrows).

We further examined the intercellular transportations of VP22-EGFP and EGFP-VP22 using BrdU labeled MSCs (Figure 6 I–P). The results also showed that both VP22-EGFP and EGFP-VP22 were detected not only in T-antigen positive COS-1 cells but also in the T-antigen negative/BrdU positive MSCs (Figure 6 L and P, arrows).

These results demonstrated the intercellular transportations of both fusion proteins between COS-1 cells and MSCs. This suggested an intriguing usage of VP22 as a delivery vehicle for the intercellular transportation of MSC modification gene products so as to enhance the therapeutic efficacy of MSC transplantation after acute myocardial infarction.

### Fusion Proteins VP22-hBcl-xl and hBcl-xl-VP22 Failed to Traffic between COS-1 Cells and MSCs

Bcl-xL belongs to the Bcl-2 protein family. It plays an important anti-apoptotic regulatory role in the cell apoptotic pathway. The adenoviral mediated expression of hBcl-xL in rat heart has been demonstrated to be able to inhibit the apoptosis of the ischemic cardiomyocytes after myocardial infarction, and the over expression of hBcl-xL could also prolong the cold preservation time of the cardiac transplants during heart transplantation [45], [46]. Thus, Bcl-xL is a good candidate for MSC anti-apoptotic modification to enhance the viability of transplanted MSCs in the deleterious ischemic microenvironment. And the fusion of VP22 to Bcl-xL might enhance the intercellular delivery of hBcl-xL in MSCs, thus enhance the efficacy of MSCs modification.

**Figure 7:**
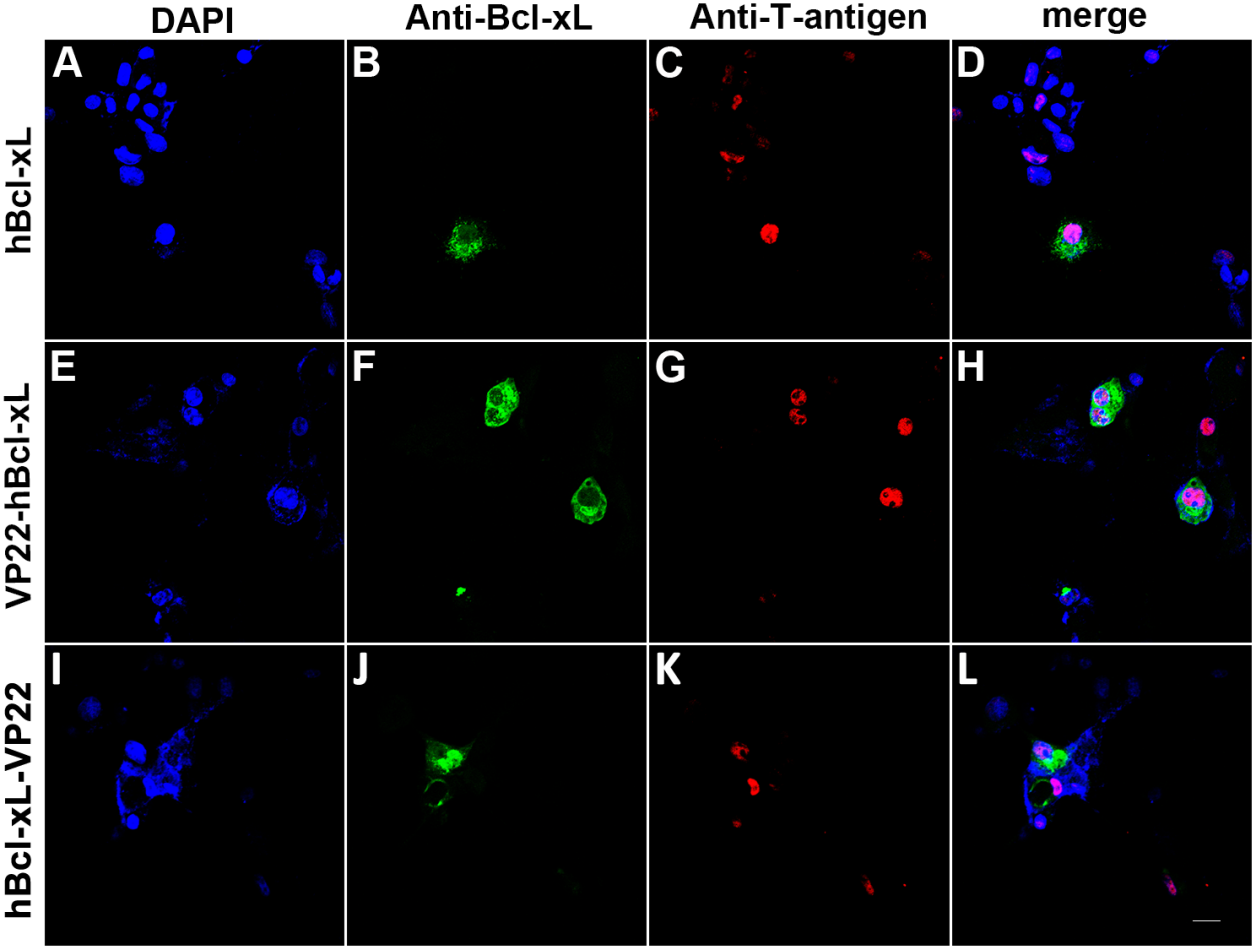
Fusion proteins VP22-hBcl-xL and hBcl-xL-VP22 failed to traffic between COS-1 cells and MSCs. COS-1 cells transfected with 0.5 µg phBcl-xL (A–D), pVP22-hBcl-xL (E–H) or phBcl-xL-VP22 (I–L) were trypsinized 24 hours after transfection and were co-plated with MSCs at a ratio of 1∶20. The co-cultured cell population were incubated for another 24 hours and were double stained with anti-Bcl-xL (B, F and J) and anti-T-antigen (C, G and K) antibodies. Bcl-xL was detected only in T-antigen positive COS-1 cells precluding the intercellular spread of VP22-hBcl-xL and hBcl-xL-VP22. Scale bar represents 20 µm.

In order to investigate this possibility, we constructed expression plasmids phBcl-xL, pVP22-hBcl-xL and phBcl-xL-VP22 for the expressions of the wild type hBcl-xL protein and the fusion proteins of VP22-hBcl-xL and hBcl-xL-VP22 (Figure 1). To determine whether VP22-hBcl-xL and hBcl-xL-VP22 could spread between COS-1 cells and MSCs, COS-1 cells were transfected with 0.5 µg phBcl-xL (Figure 7 A–D) as a negative control or with 0.5 µg pVP22-hBcl-xL (Figure 7 E–H) and phBcl-xL-VP22 (Figure 7 I–L). The transfected cells were trypsinized 24 hours later and were co-plated with untransfected MSCs at a ratio of 1∶20. The co-cultured cell populations were incubated for another 24 hours and were double stained with anti-Bcl-xL (Figure 7 B, F and J) and anti-T-antigen (Figure 7 C, G and K) antibodies. However, as in the control cells (Figure 7 D), VP22-hBcl-xL and hBcl-xL-VP22 were detected only in T-antigen positive COS-1 cells which precludes the intercellular spread of VP22-hBcl-xL and hBcl-xL-VP22 (Figure 7 H and L).

Thus, our results suggested that fusion proteins VP22-Bcl-xL and hBcl-xL-VP22 could not travel between COS-1 cells and MSCs.

### Fusion Proteins VP22-hBcl-xl and hBcl-xl-VP22 were Present in the Insoluble Fractions of the Cell Lysates

**Figure 8:**
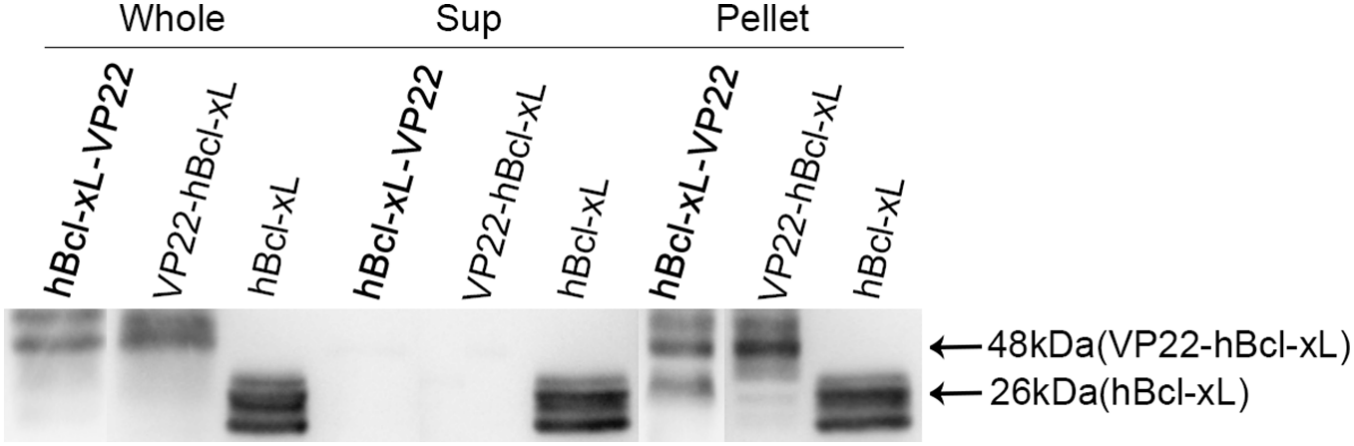
Fusion proteins VP22-hBcl-xL and hBcl-xL-VP22 are present in the insoluble fractions of the cell lysates. Cos-1 cells were harvested 48 h after transfection and lysed either in RIPA lysis buffer or in NP40 lysis buffer. The cell lysates in NP-40 lysis buffer were further sonicated and centrifuged to obtain fractions containing soluble supernatants (Sup) and insoluble pellets (Pellet). Proteins were resolved by 10% SDS-PAGE and transferred to PVDF membranes, then probed with mouse anti-myc antibody (A), rabbit anti-Bcl-xL antibody (B) and mouse anti-β-Tubulin antibody (loading control). It was shown that at least one half of the total hBcl-xL protein was present in the soluble supernatants while VP22-hBcl-xL and hBcl-xL-VP22 were absent, indicating the solubility of Bcl-xL in the absence of VP22. And, instead, VP22-hBcl-xL and hBcl-xL-VP22 were detected in the insoluble pellets of the cell lysates, indicating the interactions of the fusion proteins with other cellular components which could preclude the intercellular trafficking of the fusion proteins.

One reason of the failure of VP22-hBcl-xL and hBcl-xL-VP22 to spread between cells could be the absence of solubility of the fusion proteins resulting from the possible interactions of the fusion proteins with other cellular components, which could trap the fusion proteins inside the protein complexes and preclude their intercellular trafficking. Therefore, we transfected COS-1 cells with pVP22-hBcl-xL, phBcl-xL-VP22 or phBcl-xL expression plasmids. Forty eight hours later, we checked the expressions and distributions of VP22-hBcl-xL, hBcl-xL-VP22 and hBcl-xL in the soluble (the supernatant) and the insoluble (the pellet) fractions of the whole producer cell lysates by western blot. It was shown that, all the three target proteins were expressed and were of the expected size in producer cells. As we expected, the analysis of the supernatants revealed that at least one half of the total hBcl-xL protein was present in the soluble fraction of the cell lysate (Figure 8, Sup) indicating the solubility of Bcl-xL in the absence of VP22. And in agreement with our speculation, the fusion proteins VP22-hBcl-xL and hBcl-xL-VP22 were not detected in the supernatant fraction (Figure 8, Sup); instead, they were detected in the insoluble fraction of the cell lysates (Figure 8 Pellet). Therefore, it is highly probable that the failure of VP22-hBcl-xL and hBcl-xL-VP22 to spread between cells is due to the interactions with other cellular components resulting in the intracellular containment of the fusion proteins. Indeed, a similar observation had been reported, where the formation of protein complex impeded the intercellular trafficking of VP22 fusion proteins [49].

Another possible reason for the failed intercellular movement of VP22-hBcl-xL and hBcl-xL-VP22 is that, in this study, we fused hBcl-xL immediately to the N- or C-terminal end of VP22 without any linker amino acids in between the two fused parts. The lack of linker amino acids could lead to a three-dimensional structural interference between the two fused proteins. It was reported that the C-terminal 34 amino acids are critical for the intercellular trafficking of VP22 protein [38]. In our study, the deprivation of the intercellular trafficking of VP22 fusion proteins might result from an incorrect conformation of the extreme C-terminal region of VP22 protein. Thus, the usage of a proper linker during the fusion protein construction might help to retain the functions of both of the fused proteins.

Although, in this study, the fusion proteins VP22-hBcl-xL and hBcl-xL-VP22 failed to spread between cells, the observed intercellular transportations of fusion proteins VP22-EGFP and EGFP-VP22 still indicate the intriguing usage of VP22 as a delivery vehicle for the intercellular transportation of MSC modification gene products so as to enhance the therapeutic efficacy of MSC transplantation after acute myocardial infarction.
